# Exploring Knowledge, Attitudes, Practices, Environmental Concerns, and Barriers to Biodegradable Packaging Among University Students at Two Public Universities in Bangladesh: A Cross‐Sectional Study

**DOI:** 10.1002/hsr2.72880

**Published:** 2026-07-30

**Authors:** Razia Sultana, Jannatun Naeem, Nadi Rani Dey, Maliha Tayaba, Sultan Mahmud Imran, Nitai Roy

**Affiliations:** ^1^ Department of Post‐Harvest Technology Patuakhali Science and Technology University Patuakhali Bangladesh; ^2^ Faculty of Nutrition and Food Science Patuakhali Science and Technology University Patuakhali Bangladesh; ^3^ Department of Community Health and Hygiene Patuakhali Science and Technology University Patuakhali Bangladesh; ^4^ Department of Biochemistry and Molecular Biology Patuakhali Science and Technology University Patuakhali Bangladesh

**Keywords:** Bangladesh, biodegradable packaging, knowledge, attitudes, and practices (KAP), sustainable consumption, university students

## Abstract

**Background and Aims:**

Biodegradable packaging has gained increasing attention globally as an eco‐friendly alternative to conventional plastics with the aim of reducing environmental pollution and promoting sustainability. This study aimed to examine university students' knowledge, attitudes, and practices (KAP) along with the barriers and environmental concerns regarding biodegradable packaging in southern Bangladesh.

**Methods:**

A cross‐sectional survey was conducted between June and July 2025, involving 387 students through convenience sampling and self‐reported interviews.

**Results:**

The findings showed moderate KAP toward biodegradable packaging (54.7%, 58.9%, and 57%, respectively). Knowledge was significantly higher among participants aged 21–23 and ≥ 24 years, hostel residents, and those whose mothers had completed higher secondary education. Attitudes were higher among females, students aged 21–23, and students in advanced study years. Practices were significantly higher among females, and students in the later stages of their studies. By contrast, students from rural areas demonstrated significantly lower attitudes and practices. Additionally, the lack of exposure to biodegradable packaging in university courses, absence of formal training on environmental conservation, non‐participation in environmental volunteer work, and not following eco‐friendly social media pages exhibited lower levels of KAP. Difficulties in finding biodegradable packages in the local market were the most commonly described barriers (14.1%) among the participants. Pollution of rivers, oceans, and natural areas by plastic packaging (11.9%) was reported as the most frightening environmental danger identified by the participants.

**Conclusion:**

The results highlight that focused education, training, and increased accessibility of biodegradable packaging are essential to enhance students' KAP and overcome barriers to sustainable adoption, particularly among specific demographic groups and in rural areas of Bangladesh.

## 1 Introduction

Packaging plays a vital role in protecting and preserving food by extending shelf life [1]. Traditionally, packaging materials are made from inexpensive, petroleum‐based, non‐disposable components because they are inexpensive and possess strong barriers and mechanical properties [2]. However, because most of these materials are single‐use, their disposal poses serious environmental threats to ecosystems worldwide. Plastic production and waste generation have risen to the level of a global environmental crisis, with plastic pollution now affecting virtually all terrestrial, freshwater, and marine ecosystems and threatening ecosystem services and human health across regions and income levels [3, 4, 5]. Global plastic consumption has exceeded 700 million tons annually, with earlier estimates projected to approach 1 billion tons by 2021, although more recent data suggest somewhat lower amounts [6]. Most plastics remain nonrenewable and nonbiodegradable, with much of this waste being mismanaged or leaking into the environment. Alarmingly, the World Economic Forum (2016) predicted that by 2050, oceans could contain more plastic than fish. Recent global assessments estimate that 19–23 million metric tons of plastic entered aquatic ecosystems in 2016 and that, without transformative interventions, annual emissions to oceans could reach tens of millions of tons by 2030 and trillions of particles floating at the surface, underscoring the urgency of systemic solutions such as sustainable and biodegradable packaging [7, 8, 9].

Plastic pollution is also a growing concern in Bangladesh, where throwaway culture encourages widespread littering, particularly in urban areas. According to garbage concerns, expanding urban populations strongly correlate with increased garbage creation. Since 2005, the average daily per capita garbage generation in Bangladesh has increased faster than urban population growth [10]. In Dhaka alone, over 48% of collected plastic trash ends up in landfills, with only 37% recycled [11]. This concern has prompted researchers to investigate plastic materials created from ecologically advantageous or “environmentally friendly” polymers [12]. These national challenges mirror wider global patterns, where inadequate waste management, low recycling rates, and leakage from landfills contribute to the long‑term accumulation of macro‐ and microplastics in soils, rivers, and oceans [3, 5, 13].

Simultaneously, consumer attitudes regarding food packaging have shifted in response to growing environmental concerns. Many people view packaging as an essential component of food and garbage left behind after consumption [14]. Concerns regarding waste disposal have raised public awareness regarding the need for sustainable solutions [6]. While conventional packaging has obvious advantages, the desire for biodegradable alternatives has increased in parallel with environmental consciousness to reduce plastic waste [1]. Interest in biodegradable and bio‐based polymers has intensified as part of a broader strategy to reduce plastic emissions, support circular economy goals, and mitigate climate and pollution impacts [15, 16].

Globally, countries are increasingly integrating biodegradable packaging into their environmental policies to decrease their dependence on single‐use plastics [17, 18]. For example, the Single‐Use Plastics Directive of the European Union has established ambitious reduction objectives and promoted the use of biodegradable and compostable materials, particularly in the food packaging and retail sectors [19]. These trends are also reflected in consumer preferences, as biodegradable packaging is frequently linked to superior quality, sustainability, and value, which, in turn, affects both product selection and satisfaction [20]. The demand for sustainable packaging is particularly high among Gen‐Z consumers, as evidenced by current market trends [21].

Biodegradable polymers, which can be degraded via microbial action within a specified timeframe and environment, can be obtained through biomass, microbial extraction, biotechnological synthesis from bio‐based monomers, or conventional synthesis from artificial monomers [22]. These environmentally favorable materials undergo complete degradation into non‐toxic byproducts without accumulating in the ecosystem [23]. Despite their potential, high production costs continue to be a significant impediment to their widespread adoption in developing countries [24]. Nonetheless, global reviews emphasize that biodegradable and bio‐based packaging can play a critical role in achieving international sustainability targets if technical, economic, and infrastructural challenges are addressed through coordinated innovation and policy [13, 16].

Within this context, the Knowledge, Attitudes, and Practices (KAP) framework offers a useful lens for understanding how individuals move from awareness of an issue to sustained behavior change. In KAP models, “knowledge” refers to what people know and understand about an issue, “attitudes” capture their beliefs and feelings, and “practices” describe their actual behaviors or habits in daily life [25, 26]. KAP studies on sustainability and plastic use have shown that higher environmental knowledge tends to foster more positive attitudes, and these attitudes in turn are key predictors of pro‑environmental practices, even though the links are often imperfect and influenced by contextual barriers [25, 27, 28, 29, 30]. For example, university‑based KAP surveys frequently find high or moderate sustainability knowledge but only moderate levels of sustainable practices, revealing a persistent knowledge–action gap and highlighting the need for targeted interventions that address both attitudes and structural constraints [25, 27]. By mapping how KAP are distributed in a population, KAP studies can identify specific misconceptions, motivational levers, and behavioral obstacles, thereby informing the design of education programs, campus initiatives, and policy measures aimed at promoting sustainable consumption and plastic reduction [31, 32, 33]. In the context of biodegradable packaging, KAP‐based evidence can help determine whether low adoption is primarily driven by lack of awareness, weak pro‐environmental attitudes, or practical barriers such as limited access and cost, and thus guide more effective strategies to increase uptake [34, 35].

Bangladesh has made major advances in developing sustainable packaging solutions. It was the first country to prohibit the use of polythene shopping bags on a national scale and is one of the world's largest producers of jute [36]. In addition, innovative plastic alternatives have been created. The government banned single‐use polythene bags in grocery stores and announced plans to phase out 17 categories of plastic products in October 2024, which was more recent [37]. However, obstacles such as inadequate market adoption, high manufacturing costs, and restricted production capacity continue to impede its widespread implementation [38]. Nevertheless, these endeavors underscore the increasing recognition of the necessity for sustainable packaging, and emphasize Bangladesh's potential as a model for incorporating biodegradable solutions into national policy frameworks, particularly in low‐ and middle‐income contexts. Despite this, little is known about how future decision‑makers in Bangladesh, such as university students, understand biodegradable packaging, how they feel about it, and whether they actually use it. Existing KAP and related studies on plastics, sustainability, and packaging have largely focused on other countries and topics, such as sustainable consumption in Malaysia, sustainable development in Tibet, or single‑use plastic behaviors in the Philippines and South Africa [25, 27, 34, 39]. To date, there appears to be no KAP study specifically examining biodegradable packaging among Bangladeshi university students, leaving a critical gap in evidence to support behavior‑change interventions and policy design in this area.

Given these considerations, this study aimed to investigate university students' KAP, impediments, and environmental concerns regarding biodegradable packaging in southern Bangladesh. We hope to discover gaps, behavioral impediments, and intervention opportunities by assessing their knowledge, perceptions, and concerns. Finally, the findings of this study may help policymakers, industries, and educational institutions to promote sustainable consumption patterns and accelerate the use of eco‐friendly packaging solutions in Bangladesh and elsewhere.

## 2 Methodology

### 2.1 Study Settings

This cross‐sectional study was conducted at two state‐owned universities in Bangladesh: Patuakhali Science and Technology University (PSTU) and University of Barishal (BU). Both universities are located in the southern coastal region, which is particularly sensitive to environmental degradation and climate change. Students at these universities come from various socioeconomic and geographic backgrounds, including rural, semi‐urban, and urban locations. PSTU has specialized programs in agriculture, nutrition and food sciences, and environmental sciences, providing students with more opportunities to learn about environmental sustainability, new food packaging solutions, and effective waste disposal. By contrast, BU is located in a fast‐developing urban center; thus, students are more likely to encounter urban consumer practices and environmental campaigns. These complementary environments make the two colleges ideal for gathering a diverse variety of KAP about biodegradable packaging.

### 2.2 Study Design and Sampling

This exploratory study was designed to investigate students' baseline awareness, and as such, it did not test formal hypotheses. A cross‐sectional survey was conducted between June and July 2025 with a structured self‐administered questionnaire to measure students' KAP, environmental concerns, and impediments to biodegradable packaging. A total of 387 valid replies were included in the final analysis (PSTU: 200/387 [51.7%]; BU: 187/387 [48.3%]). Eligible participants were Bangladeshi male and female students aged ≥ 18 years and enrolled in bachelor's or master's programs at the specified universities. A convenience sampling method was used in this study. Data collection took place in multiple settings within the universities, including general campus areas, student dormitories, and classrooms. Students who declined or submitted questionnaires with any missing item were excluded from the analysis.

### 2.3 Sample Size Calculation

The sample size was calculated using Cochran's formula for a finite population. First, the initial sample size was estimated using *n*_0_ = *Z* ^2^pq/*e* ^2^, where *Z* = 1.96 at a 95% confidence level, *p* = 0.5, q = 1 − *p*, and *e* = 0.05 margin of error, yielding an initial sample size of 384. Since the total population of undergraduate and postgraduate students in the two universities was finite (*N* = 13,000; PSTU = 3,000 and BU = 10,000), the finite population correction was applied, resulting in a corrected sample size of approximately 373. To account for potential non‐response and incomplete questionnaires, a 5%–10% adjustment was considered, resulting in a target sample size of approximately 392–410. A total of 394 responses were initially collected; after data cleaning, 387 valid responses were included in the final analysis. Although slightly below the adjusted target, the final sample still exceeds the minimum required size after finite population correction (*n* = 373).

### 2.4 Interviews and Data Collection

Data were collected through self‐report interviews using a structured questionnaire (Supporting Information: Supplement 1 and Supplement 2). The questionnaire was developed following a comprehensive review of the relevant literature [1, 6, 40]. The questionnaire was originally developed in English, translated into Bangla (the local language), and back‐translated into English by a separate bilingual researcher to ensure linguistic equivalence and fidelity. A pilot test was conducted with a convenience sample of 15 students from two universities and adjustments were made based on their feedback [41]. Three interviewers were responsible for data collection, and the interviews were arranged at times convenient for the participants. Written consent was obtained from all individuals who agreed to participate. Each interview lasted approximately 12–15 min.

### 2.5 Study Variables and Measures

#### 2.5.1 Socio‐Demographic Factors, Socio‐Economic, and Environmental Exposure Variables

The sociodemographic data gathered encompassed several variables, such as gender, age, educational background, religion, residential status, employment status of family members, and monthly household income. Additionally, exposure‑related variables included previous participation in environmental seminars or workshops, academic exposure to biodegradable packaging, involvement in volunteer environmental activities, engagement with social media and digital platforms related to eco‐friendly products, and their primary source of information about biodegradable packaging.

### 2.6 Students' Knowledge Towards Biodegradable Packaging

Participants' knowledge of biodegradable packaging was assessed using a structured set of 12 closed‐ended questions, “each offering three possible answers: ‘Yes’, ‘No', and ‘Not sure'. Correct answers typically received 1 point for a “Yes” response, while incorrect answers (“No” or “Not sure”) were assigned 0 points. However, for specific questions (numbers 4, 6, and 12), a “No” or ‘Not sure’ response was considered correct and awarded 1 point, with “Yes” responses given 0 points. The total knowledge score is the sum of points across all 12 questions, with a higher total score indicating higher knowledge. The internal consistency of the knowledge items was evaluated using Cronbach's alpha, which yielded a coefficient of 0.721, indicating an acceptable level of reliability.

### 2.7 Students' Attitude Towards Biodegradable Packaging

Participants' attitudes toward biodegradable packaging were assessed using 14 statements. Among the issues discussed in these statements, trust, environmental concerns, purchasing decisions, and health maintenance are paramount. Each statement was answered with five alternative responses: strongly agree, agree, neutral, disagree, and strongly disagree. All responses were rated on a 1‐5 scale. For positively worded statements, 1 corresponds to strongly disagree and 5 to strongly agree. For negatively worded statements, the scale was reversed (1 = strongly agree, 5 = strongly disagree) to maintain consistency in interpretation (i.e., a higher score reflected a more positive attitude toward biodegradable packaging). The total attitude score was the sum of the points across all 14 statements. The internal consistency of the attitude items was evaluated using Cronbach's alpha, which yielded a coefficient of 0.807, indicating a good level of reliability.

### 2.8 Students' Practice of Biodegradable Packaging

Numerous aspects, such as the use of recyclable bags, choice of biodegradable packaging, reuse of materials, ensuring product labels, and proper waste disposal, are covered by this segment. They also emphasize community activities, such as talking about sustainable packaging with people, their involvement in environmental events, and the creation of awareness through social media. A set of 15 questions were offered, and practices were rated on an ordinal scale of 1 to 5 (never = 1, rarely = 2, sometimes = 3, often = 4, always = 5). Only four questions were marked in reverse (items 8, 10, 13, and 14) and the questions where “Never,” “Rarely, Sometimes, Often and Always were taken as 5 points, 4 points, 3 points, 2 points and 1 point, respectively. The total practice score was the sum of the points across all 15 questions. The internal consistency of the practice items was evaluated using Cronbach's alpha, which yielded a coefficient of 0.713, indicating an acceptable level of reliability.

### 2.9 Barriers and Environmental Concerns of Adopting Biodegradable Packaging

Barriers to the adoption of biodegradable packaging were assessed using a multiple‐response question where participants were asked to select all applicable constraints they perceived in using biodegradable packaging. Respondents could indicate more than one barrier, including higher cost compared to plastic packaging (economic constraint), limited availability in local markets and inadequate waste management systems (infrastructural constraints), lack of knowledge and awareness about biodegradable packaging and its proper disposal (informational constraints), and consumer perceptions such as uncertainty about quality, lack of durability, unattractive design, and social or cultural preference for traditional packaging (behavioral and perceptual constraints).

Similarly, environmental concerns were measured using a multiple‐response item in which participants identified the environmental issues they associate with conventional packaging. These items captured perceptions related to pollution, climate change, resource depletion, waste management challenges, and ecological and health impacts. Participants were allowed to select multiple concerns based on their understanding and awareness.

### 2.10 Ethical Approval and Consent to Participate

This study was conducted in accordance with the ethical principles outlined in the Declaration of Helsinki (2013). Prior to data collection, ethical clearance was obtained from the appropriate institutional authorities. Specifically, ethical approval was granted by the Institutional Ethical Committee of Patuakhali Science & Technology University (Approval No. PSTU/IEC/2025/20 (iii)). Participation in the study was entirely voluntary. All participants were informed about the purpose and procedures of the research, and informed consent was obtained before administering the survey. Respondents were assured of the confidentiality and anonymity of their information, and no personally identifiable data were collected. Participants were also informed of their right to withdraw from the study at any time without any consequences.

### 2.11 Statistical Analyses

All statistical analyses were performed using IBM SPSS Statistics, version 27.0 (IBM Corp., Armonk, NY, USA). Two‑sided tests were used throughout, and a priori statistical significance was set at (*p* < 0.05). Continuous variables (e.g., total knowledge, attitude, and practice scores) were first explored using descriptive statistics (mean, SD, minimum, and maximum) and visually inspected using histograms and *Q–Q* plots, consistent with recommendations to examine distributional assumptions before choosing parametric or non‑parametric tests [42]. The Shapiro–Wilk test was applied to formally assess normality of continuous variables; a (*p* > 0.05) was interpreted as consistent with normal distribution [43, 44]. As the KAP scores showed non‑normal distributions (Shapiro–Wilk (*p* < 0.05)), non‑parametric tests were used for group comparisons [45]. To compare KAP scores between two groups (e.g., male vs. female), we used the Mann–Whitney *U* test [46]. For comparisons across variables with more than two categories (e.g., education level, residence type), we used the Kruskal–Wallis H test [45].

To examine the associations between sociodemographic/exposure factors and KAP outcomes, we fitted multivariable linear regression models with total KAP scores as dependent variables and all pre‑specified sociodemographic and exposure factors entered simultaneously as independent variables. Before model fitting, assumptions of linear regression (linearity, homoscedasticity, normality of residuals, and multicollinearity) were evaluated through histogram and *Q–Q* plots, scatterplots, and variance inflation factors (VIF), in keeping with recommendations for diagnosing multicollinearity using VIF and related indices [47, 48, 49]. Regression results are reported as standardized beta coefficients (β) with their corresponding 95% confidence intervals (CI) and (*p*) values. No data transformation was attempted for score variables because non‑parametric tests and regression models accompanied by explicit multicollinearity diagnostics provide appropriate inference for ordinal or skewed data without assuming strict normality [47].

## 3 Results

**Figure 1:**
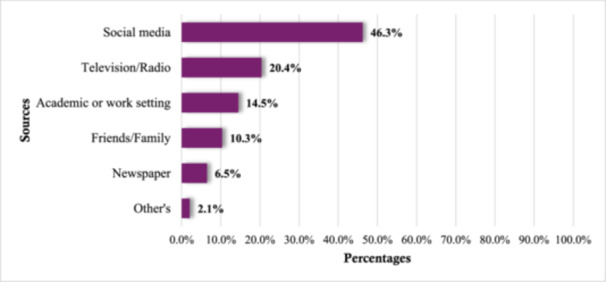
Source of information about biodegradable packaging.

In this study, 387 Bangladeshi university students participated; among them, 62.5% were female and 56.8% of students were between the ages between 21 and 23 years. The majority of participants (82.9%) were identified as Muslims. 37.2% of them had attained a B. Sc. 1st Year; more than three‐quarters (75.5%) of them are currently living in a hostel (with friends/roommates). Regarding permanent residence, approximately 42.9% residents lived in rural areas. In terms of the father's education level, 39.3% had a bachelor's degree or above, while 31.3% of their mothers had completed a secondary level of education. 41.9% Of participants' fathers were employed, whereas 87.3% of their mothers were unemployed/retired. personal monthly costs of the majority of participants (72.9%) were in the range of 5000–10,000 BDT. Of the total respondents, the environmental seminar was attended by 52.2%, while 50.6% didn't learn about biodegradable packaging in university courses. and Environmental volunteer work was done by 59.4% of participants, while 50.9% had not received any formal education (Table 1). Social media was the most influential platform, with nearly half of the respondents (46.3%) which indicated it as their initial source of awareness about biodegradable packaging. The second most sources of biodegradable packaging were television and radio, accounting for 20.4%, while academic or work settings contributed 14.5%. About 10.3% participants identified from friends and family, and only 6.5% reported newspapers as their source (Figure 1).

**Table 1:** Sociodemographic characteristics of the participants (*N* = 387).

Variables	Category	Frequency	Percentage
Sociodemographic and socioeconomic characteristics			
Age (in years)	18–20 years	81	20.9%
	21–23 years	220	56.8%
	24 years and above	86	22.2%
Gender	Male	145	37.5%
	Female	242	62.5%
Religion	Islam	321	82.9%
	Hinduism	63	16.3%
	Other's^a^	3	0.8%
Current educational level	B. Sc. 1st year	144	37.2%
	B. Sc.2nd year	73	18.9%
	B. Sc. 3rd year	51	13.2%
	B. Sc. 4th year	74	19.1%
	M. Sc./MS	45	11.6%
Current living arrangement	With family	34	8.8%
	Hostel (with friends/roommates)	292	75.5%
	Mess/rented house with others	61	15.8%
Permanent residence	Urban	153	39.5%
	Semi‐urban	68	17.6%
	Rural	166	42.9%
Father's education level	No formal education	28	7.2%
	Primary	47	12.1%
	Secondary	76	19.6%
	Higher secondary	84	21.7%
	Bachelor's or above	152	39.3%
Mother's education level	No formal education	22	5.7%
	Primary	64	16.5%
	Secondary	121	31.3%
	Higher secondary	103	26.6%
	Bachelor's or above	77	19.9%
Father's employment status	Employed	162	41.9%
	Unemployed	13	3.4%
	Retired	65	16.8%
	Business	147	38.0%
Mother's employment status	Employed	49	12.7%
	Unemployed/retired	338	87.3%
Personal monthly cost	< 5000 BDT	99	25.6%
	5000–10,000 BDT	282	72.9%
	> 10,000 BDT	6	1.6%
Environmental exposure‐related characteristics			
Participant in environmental seminars/workshops	Yes	202	52.2%
	No	185	47.8%
Learned about biodegradable packaging in university courses	Yes	191	49.4%
	No	196	50.6%
Participate in a cleanliness drive or environmental volunteer work	Yes	230	59.4%
	No	157	40.6%
Received any formal education or training on environmental conservation	Yes	197	50.9%
	No	190	49.1%
Following any social media pages or influencers promoting eco‐friendly products	Yes	210	54.3%
	No	177	45.7%

^a^ Included Christianity and Buddhism.

In Table 2, the respondents presented varying levels of knowledge about biodegradable packaging, with an overall correct response rate of 54.7% (6.56/12 × 100). Most of the participants (82.4%) heard the term “biodegradable packaging,” and two‐thirds (71.3%) of them knew that biodegradable packaging breaks down naturally in the environment, demonstrating a relatively high level of baseline knowledge. Approximately 53.7% respondents could identify biodegradable packaging while shopping at a store. However, significant gaps were noted in practical knowledge, with only 29.2% being familiar with any symbols or certifications that indicate biodegradable packaging. Several misconceptions were found in the negatively phrased items, such as 64.1% respondents believing that biodegradable packaging is harmful to the environment, 65.1% believing that biodegradable packaging takes longer to decompose than plastic, and 37.0% believing that biodegradable packaging cannot be made from traditional materials like banana leaves or earthen pots. There was a serious misunderstanding indicated by the lowest correct response rate (18.3%), in all products labeled “biodegradable” decomposed quickly in any environment (Table 2).

**Table 2:** Knowledge toward biodegradable packaging (*N* = 387).

Items	Statements (α = 0.721)	Mean ± SD	Yes (%)^b^
K1	Have you ever heard the term “biodegradable” packaging?	0.82 ± 0.381	319 (82.4)
K2	Are you aware that biodegradable packaging is often made from plant‐based materials like cornstarch or cellulose?	0.68 ± 0.465	265 (68.5)
K3	Can you identify biodegradable packaging when shopping at a store?	0.54 ± 0.499	208 (53.7)
K4	Biodegradable packaging is harmful to the environment?^a^	0.64 ± 0.480	248 (64.1)
K5	Do you know that biodegradable packaging breaks down naturally in the environment?	0.71 ± 0.453	276 (71.3)
K6	Biodegradable packaging takes longer to decompose than plastic.^a^	0.65 ± 0.477	252 (65.1)
K7	Do you know that biodegradable packaging can be composted under suitable conditions?	0.54 ± 0.499	210 (54.3)
K8	Are you familiar with any symbols or certifications that indicate biodegradable packaging?	0.29 ± 0.455	113 (29.2)
K9	Do you know that using biodegradable materials supports environmental sustainability goals?	0.66 ± 0.475	255 (65.9)
K10	Do you know that biodegradable packaging can sometimes cost more than plastic packaging?	0.56 ± 0.498	215 (55.6)
K11	All products labeled “biodegradable” decompose quickly in any environment.^a^	0.18 ± 0.388	71 (18.3)
K12	Biodegradable packaging cannot be made from traditional materials like banana leaves or earthen pots.^a^	0.37 ± 0.483	143 (37.0)

*Note:* α = Cronbach ɑ reliability coefficient for the knowledge scale.

K1–K12 = individual knowledge items assessing participants’ knowledge toward biodegradable packaging, where K1–K12 represent specific questionnaire statements, as listed in the table.

^a^ Statements are reversely coded.

^b^ Correct response to the statements.

Significant variations in the mean score for biodegradable packaging knowledge were observed across various sociodemographic factors such as age, educational level, religion, place of residence, family monthly income, and educational qualification of parents (Table 5).

The results of the linear regression analysis of the factors associated with knowledge of biodegradable packaging are presented in Table 6. Age was a significant factor, as participants aged 21‐23 years (β = 0.152) and 24 years and above (β = 0.203) had significantly higher knowledge scores than those aged 18‐20 years. Participants' living status played an important role, as those who resided in hostels with friends or roommates had greater knowledge (β = 0.138) than those living with families. Additionally, participants whose mothers had completed higher secondary education showed significantly better knowledge (β = 0.285) than those with no formal education (β = 0.045) or primary education (β = 0.142). Students who had not learned about biodegradable packaging in their university courses scored lower (β = −0.237). Similarly, not receiving any formal education or training on environmental conservation was associated with lower knowledge of biodegradable packaging (β = −0.121). Lack of participation in social media pages or influencers promoting eco‐friendly products was also associated with lower knowledge levels (β = −0.233). Other factors, such as gender, religion, father's education, and monthly personal costs, did not show statistically significant effects. All reported associations were considered statistically significant at *p* < 0.05.

The respondent's level of attitude toward biodegradable packaging, shown in Table 3, demonstrates that the overall correct response rate for the attitude test was 58.9% (41.20/70 × 100). Most of the respondents, about 42.6%) strongly agreed that they were concerned about plastic pollution in Bangladesh, and 37.0% strongly agreed that they encouraged others to use biodegradable packaging. 36.4% of them agreed that they cared about the environmental impact of packaging when shopping and 33.3% agreed that using biodegradable packaging should be a habit for everyone. There was a mixed response spotted to be willingness to pay more; 43.2% agreed or strongly agreed that they were not willing to pay more for products with biodegradable packaging, and 34.6% were neutral about their responsibility for choosing products with biodegradable packaging. Most of them (35.1%) strongly disagreed that using biodegradable packaging does not contribute to a sustainable future, and 39.0% disagreed that it is the only trend that will not grow in the future, and also 35.1% disagreed that it is not visually appealing or attractive. Only 29.5% of respondents agreed that industries should drive the adoption of biodegradable packaging, signifying a relatively low level of support for industry‐led (Table 3).

**Table 3:** Attitudes toward biodegradable packaging (*N* = 387).

Items	Statements (α = 0.807)	Mean ± SD	Strongly disagree	Disagree	Neutral	Agree	Strongly agree
A1	I care about the environmental impact of packaging when shopping.	3.60 ± 1.210	39 (10.1)	23 (5.9)	88 (22.7)	141 (36.4)	96 (24.8)
A2	I am concerned about plastic pollution in Bangladesh.	3.95 ± 1.267	41 (10.6)	13 (3.4)	35 (9.0)	133 (34.4)	165 (42.6)
A3	I prefer eco‐friendly packaging even if the price is high.	2.98 ± 1.366	64 (16.5)	102 (26.4)	66 (17.1)	86 (22.2)	69 (17.8)
A4	I believe using biodegradable packaging cannot reduce health risks.^a^	2.82 ± 1.134	55 (14.2)	97 (25.1)	122 (31.5)	87 (22.5)	26 (6.7)
A5	Eco‐friendly packaging is not visually appealing and attractive.^a^	2.50 ± 1.064	69 (17.8)	136 (35.1)	119 (30.7)	44 (11.4)	19 (4.9)
A6	I feel responsible for choosing products with biodegradable packaging.	3.14 ± 1.104	33 (8.5)	71 (18.3)	134 (34.6)	107 (27.6)	42 (10.9)
A7	I am not willing to pay more for products with biodegradable packaging.^a^	3.11 ± 1.327	60 (15.5)	73 (18.9)	87 (22.5)	99 (25.6)	68 (17.6)
A8	It is not necessary to make Biodegradable packaging mandatory in Bangladesh.^a^	2.39 ± 1.314	123 (31.8)	117 (30.2)	69 (17.8)	28 (7.2)	50 (12.9)
A9	I believe industries should take the lead in adopting biodegradable packaging	2.85 ± 1.242	57 (14.7)	109 (28.2)	107 (27.6)	63 (16.3)	51 (13.2)
A10	I believe biodegradable packaging is a trend that will not grow in the future.^a^	2.37 ± 1.152	91 (23.5)	151 (39.0)	83 (21.4)	33 (8.5)	29 (7.5)
A11	I encourage others to use biodegradable packaging.	3.84 ± 1.189	24 (6.2)	32 (8.3)	69 (17.8)	119 (30.7)	143 (37.0)
A12	Students should be educated about biodegradable packaging.	2.78 ± 1.289	69 (17.8)	119 (30.7)	77 (19.9)	74 (19.1)	48 (12.4)
A13	Using biodegradable packaging does not contribute to a sustainable future.^a^	2.10 ± 1.120	136 (35.1)	139 (35.9)	72 (18.6)	16 (4.1)	24 (6.2)
A14	Using biodegradable packaging should be a habit for everyone.	3.58 ± 1.125	26 (6.7)	30 (7.8)	113 (29.2)	129 (33.3)	89 (23.0)

*Note:* α = Cronbach's alpha reliability coefficient for the attitude scale.

A1–A14 = individual attitude items assessing participants’ attitudes toward biodegradable packaging, where A1–A14 represent specific questionnaire statements, as listed in the table.

^a^ Statements are reversely coded.

The mean score of attitudes toward biodegradable packaging varied significantly across several sociodemographic factors, including age, level of education, living status, learning about biodegradable packaging in academia, engagement in volunteer environmental work, participation in social media, and digital platforms related to eco‐friendly products (Table 5).

In Table 6, the regression analysis suggests that students aged between 21 and 23 years showed more positive attitudes compared to the students in the 18–20 age group (β = 0.144), while students in the B. Sc. 3rd Year (β = 0.107), B. Sc. 4th year (β = 0.156) and M. Sc./MS (β = 0.196) also exhibited more positive attitudes towards biodegradable packaging than 1st Year students.

However, students from rural areas had significantly lower attitude scores (β = −0.238), and semi‐urban participants exhibited a negative attitude. It is important to note that students who did not learn about biodegradable packaging in a university course (β = −0.127) had no association with environmentally allied voluntary work (β = −0.145). Additionally, students who did not follow eco‐friendly influencers or pages (β = −0.137) had significantly lower attitude scores. All reported associations were considered statistically significant at *p* < 0.05.

The respondent's level of practice toward biodegradable packaging, as Table 4 shows, with the overall correct response rate for the practice test being 57% (42.74/75 × 100). The behavior that was most commonly employed was disposing of packaging waste responsibly, with 55.8% respondents often or strongly agreeing with this practice. Approximately 60.5% respondents reported that they sometimes or often made an effort to educate themselves on biodegradable packaging practices. Approximately 32.6% participants sometimes reused packaging materials instead of throwing them away, and 36.2% respondents sometimes chose food delivery services that offer biodegradable containers. However, participation in environmental activities related to sustainable packaging and posting on social media or online platforms to raise awareness about sustainable packaging among respondents was relatively low, with 32.6% and 31.5% of participants reporting that they rarely engaged in such activities. A significant proportion of respondents (46.3%) strongly agreed or often preferred plastic packaging because it was more convenient. About 49.4% and 46.5% participants also reported that they strongly agreed or often believed their personal choices don't impact packaging waste issues and found sustainable packaging options too difficult to use (Table 4).

**Table 4:** Practices toward biodegradable packaging (*N* = 387).

Items	Statements(α = 0.713)	Mean ± SD	Never	Rarely	Sometimes	Often	Always
P1	I carry a reusable bag to avoid plastic bags while shopping.	2.67 ± 1.119	66(17.1)	104(26.9)	132 (34.1)	61 (15.8)	24 (6.2)
P2	I look for biodegradable packaging when buying food or products.	2.71 ± 1.063	48 (12.4)	125 (32.3)	128 (33.1)	64 (16.5)	22 (5.7)
P3	I reuse packaging materials instead of throwing them away.	2.81 ± 1.113	56 (14.5)	94 (24.3)	126 (32.6)	90 (23.3)	21 (5.4)
P4	I check product labels to see if the packaging is biodegradable.	2.61 ± 1.206	78 (20.2)	116 (30.0)	104 (26.9)	55 (14.2)	34 (8.8)
P5	I dispose of packaging waste responsibly.	3.52 ± 1.201	29 (7.5)	50 (12.9)	92 (23.8)	123 (31.8)	93 (24.0)
P6	I talk to friends or family about using biodegradable packaging.	2.76 ± 1.085	55 (14.2)	97 (25.1)	142 (36.7)	71 (18.3)	22 (5.7)
P7	I participate in environmental activities related to sustainable packaging.	2.51 ± 1.049	72 (18.6)	126 (32.6)	123 (31.8)	53 (13.7)	13 (3.4)
P8	I prefer plastic packaging because it is more convenient.^a^	3.34 ± 1.190	26 (6.7)	74 (19.1)	108 (27.9)	101 (26.1)	78 (20.2)
P9	I choose food delivery services that offer biodegradable containers.	2.85 ± 1.127	50 (12.9)	94 (24.3)	140 (36.2)	69 (17.8)	34 (8.8)
P10	I believe my personal choices don't impact packaging waste issues.^a^	3.40 ± 1.184	28 (7.2)	60 (15.5)	108 (27.9)	111 (28.7)	80 (20.7)
P11	I make an effort to educate myself on biodegradable packaging practices.	3.05 ± 1.086	33 (8.5)	82 (21.2)	144 (37.2)	90 (23.3)	38 (9.8)
P12	I post on social media or online platforms to raise awareness about sustainable packaging.	2.42 ± 1.092	90 (23.3)	122 (31.5)	110 (28.4)	51 (13.2)	14 (3.6)
P13	I find sustainable packaging options too difficult to use.^a^	3.39 ± 1.174	27 (7.0)	57 (14.7)	123 (31.8)	98 (25.3)	82 (21.2)
P14	I choose products based on price, not packaging sustainability.^a^	3.10 ± 1.247	50 (12.9)	71 (18.3)	116 (30.0)	89 (23.0)	61 (15.8)
P15	I attend university seminars, workshops, or campaigns related to packaging sustainability.	2.47 ± 1.075	84 (21.7)	110 (28.4)	138 (35.7)	37 (9.6)	18 (4.7)

*Note:*α = Cronbach's alpha reliability coefficient for the practice scale.

P1–P15 = individual practice items assessing participants’ practices toward biodegradable packaging, where P1–P15 represent specific questionnaire statements, as listed in the table.

^a^ Statements are reversely coded.

The mean score for the practice of biodegradable packaging varied significantly across several sociodemographic factors, including age, educational level, present living status, academic learning about biodegradable packaging, engagement in volunteer environmental work, involvement in social media, and digital channels for eco‐friendly products (Table 5).

**Table 5:** Relationship between sociodemographic characteristics and participant knowledge, attitudes, and practices about biodegradable packaging.

**Variables**	**Knowledge**		**Attitude**		**Practice**	
	**Mean ± SD**	***p*** **value**	**Mean ± SD**	***p*** **value**	**Mean ± SD**	***p*** **value**
Age of the participant						
18–20 years	5.07 ± 2.823	**< 0.001**	36.02 ± 11.381	**< 0.001**	40.11 ± 7.265	**< 0.001**
21–23 years	6.81 ± 2.531		43.02 ± 6.807		43.80 ± 6.886	
24 years and above	7.73 ± 2.645		45.14 ± 9.233		46.45 ± 8.412	
Gender						
Male	6.21 ± 2.784	**0.028** a	39.99 ± 8.458	**< 0.001** a	41.19 ± 6.573	**< 0.001** a
Female	6.92 ± 2.719		43.25 ± 9.066		45.07 ± 7.819	
Religious						
Islam	6.65 ± 2.739	0.732	41.98 ± 8.891	0.558	43.52 ± 7.611	0.712
Hinduism	6.62 ± 2.926		42.11 ± 10.095		44.16 ± 7.673	
Other's b	8.00 ± 1.732		46.00 ± 5.568		42.33 ± 7.234	
Current education level						
B. Sc. 1st year	5.57 ± 2.737	**< 0.001**	37.35 ± 9.979	**< 0.001**	39.97 ± 6.787	**< 0.001**
B. Sc. 2nd year	6.96 ± 2.195		41.84 ± 6.612		43.70 ± 5.990	
B. Sc. 3rd Year	6.47 ± 2.452		44.31 ± 4.111		44.25 ± 6.076	
B. Sc. 4th year	7.46 ± 2.623		45.31 ± 6.178		46.05 ± 7.144	
M. Sc./MS	8.51 ± 2.785		49.33 ± 9.637		50.44 ± 8.390	
Current residence						
With family	5.82 ± 3.000	0.087	43.76 ± 8.068	0.920	45.21 ± 6.901	0.579
Hostel (with friends/roommates)	6.80 ± 2.709		41.76 ± 9.138		43.52 ± 7.778	
Mess/rented house with others	6.39 ± 2.818		42.34 ± 9.263		43.21 ± 7.121	
Permanent residence						
Urban	7.27 ± 2.742	**0.003**	46.10 ± 7.433	**< 0.001**	47.20 ± 7.642	**< 0.001**
Semi‐urban	6.46 ± 2.919		42.10 ± 7.517		42.75 ± 6.810	
Rural	6.16 ± 2.615		38.24 ± 9.413		40.67 ± 6.454	
Father's educational qualification						
No formal education	5.68 ± 3.068	**0.014**	39.57 ± 9.061	0.331	41.50 ± 8.535	0.379
Primary	6.83 ± 2.496		41.45 ± 9.177		43.96 ± 7.791	
Secondary	5.92 ± 2.827		42.66 ± 10.299		43.24 ± 8.093	
Higher secondary	7.10 ± 2.691		42.00 ± 7.883		44.30 ± 6.739	
Bachelor's or above	6.90 ± 2.708		42.36 ± 9.022		43.72 ± 7.588	
Mother's educational qualification						
No formal education	5.50 ± 2.824	**0.031**	39.91 ± 10.221	0.680	42.45 ± 9.267	0.353
Primary	6.16 ± 2.891		42.14 ± 9.610		43.56 ± 8.284	
Secondary	6.47 ± 2.933		42.50 ± 9.424		43.59 ± 7.476	
Higher secondary	7.11 ± 2.589		41.16 ± 9.106		42.95 ± 7.524	
Bachelor's or above	7.08 ± 2.432		42.97 ± 7.545		44.94 ± 6.785	
Father's employment status						
Employed	6.80 ± 2.689	**0.042**	42.54 ± 8.539	0.775	43.80 ± 6.963	0.150
Unemployed	7.77 ± 1.922		45.00 ± 8.670		46.38 ± 7.698	
Retired	7.11 ± 2.641		41.49 ± 9.832		44.60 ± 8.801	
Business	6.20 ± 2.897		41.44 ± 9.316		42.73 ± 7.657	
Mother's employment status						
Employed	7.16 ± 2.569	0.342	44.00 ± 8.354	0.249	44.49 ± 6.332	0.510
Unemployed/retired	6.58 ± 2.784		41.74 ± 9.141		43.49 ± 7.771	
Personal monthly cost						
<5000 BDT	6.35 ± 2.678	0.181	41.54 ± 9.787	0.399	43.04 ± 7.746	0.467
5000–10,000 BDT	6.79 ± 2.772		42.23 ± 8.881		43.83 ± 7.597	
>10,000 BDT	5.17 ± 3.312		40.50 ± 5.010		43.33 ± 5.888	
Participant in environmental seminars/workshops						
Yes	7.61 ± 2.572	**< 0.001** a	45.65 ± 6.511	**< 0.001** a	47.10 ± 7.060	**< 0.001** a
No	5.61 ± 2.583		38.08 ± 9.799		39.82 ± 6.246	
Learned about biodegradable packaging in university courses						
Yes	8.05 ± 2.314	**< 0.001** a	46.39 ± 6.448	**< 0.001** a	47.60 ± 6.938	**< 0.001** a
No	5.29 ± 2.469		37.78 ± 9.239		39.74 ± 6.076	
Participate in a cleanliness drive or environmental volunteer work						
Yes	7.71 ± 2.363	**< 0.001** a	45.68 ± 6.129	**< 0.001** a	46.85 ± 6.770	**< 0.001** a
No	5.10 ± 2.568		36.68 ± 9.991		38.88 ± 6.144	
Received any formal education or training on environmental conservation						
Yes	7.90 ± 2.373	**< 0.001** a	45.88 ± 6.793	**< 0.001** a	47.29 ± 7.032	**< 0.001** a
No	5.36 ± 2.539		38.04 ± 9.410		39.81 ± 6.172	
Following any social media pages or influencers promoting eco‐friendly products						
Yes	7.95 ± 2.257	**< 0.001** a	45.83 ± 6.259	**< 0.001** a	46.74 ± 7.039	**< 0.001** a
No	5.12 ± 2.512		37.51 ± 9.797		39.91 ± 6.518	
Total	6.56 ± 2.857		41.20 ± 10.694		42.74 ± 9.689	

*Note:* Bold values indicate statistical significance.

^a^ Mann–Whitney *U* test was conducted unless otherwise stated, and the Kruskal–Wallis *H* test was performed.

^b^ Included Christianity and Buddhism.

Linear regression analysis indicated that female students were more likely to engage in positive behavior when using biodegradable packaging (β = 0.153) than male students at both universities. Academically, senior students scored higher in the 4th year (β = 0.139) and M. Sc./MS (β = 0.207) than second‐year (β = 0.041) or third‐year students (β = 0.080). Participants from rural areas had notably lower practice levels (β = −0.205), compared to those from semi‐urban regions (β = −0.110). Additionally, lack of experience in university coursework (β = −0.146), non‐participation in environmental volunteer work (β = −0.132), absence of proper environmental learning (β = −0.116), and not following social media focusing on sustainable packaging materials (β = −0.112) were significantly associated with lower practice scores (Table 6). All reported associations were considered statistically significant at *p* < 0.05.

**Table 6:** Association between socio‐demographic characteristics and participant knowledge, attitudes, and practices about biodegradable packaging.

**Variables**	**Knowledge**				**Attitude**				**Practice**				**VIF**
	β	95% CI		*p* value	β	95% CI		*p* value	β	95% CI		*p*‐value	
		LL	UL			LL	UL			LL	UL		
(Constant)		4.168	7.507	< 0.001		40.506	51.252	**< 0.001**		44.129	52.889	< 0.001	
Age of the participant													
18–‐20 years	[Reference]				[Reference]								
21–23 years	0.152	0.188	1.501	**0.012**	0.144	0.524	4.750	**0.015**	−0.027	−2.132	1.314	0.641	2.303
24 years and above	0.203	0.349	2.351	**0.008**	0.010	−3.008	3.434	0.897	−0.094	−4.334	0.917	0.202	3.770
Gender													
Male	[Reference]				[Reference]								
Female	0.022	−0.391	0.641	0.634	0.098	0.172	3.493	**0.031**	0.153	1.039	3.747	**0.001**	1.358
Religious													
Islam	[Reference]				[Reference]								
Hinduism	−0.010	−0.685	0.537	0.813	0.010	−1.730	2.203	0.813	0.029	−1.002	2.204	0.461	1.108
Other's a	0.036	−1.438	3.676	0.390	0.041	−3.987	12.472	0.311	−0.001	−6.785	6.632	0.982	1.095
Current education level													
B. Sc. 1st year	[Reference]				[Reference]								
B. Sc. 2nd Year	0.030	−0.502	0.923	0.561	0.020	−1.833	2.754	0.693	0.041	−1.077	2.662	0.405	1.693
B. Sc. 3rd Year	−0.046	−1.162	0.412	0.349	0.107	0.336	5.400	**0.027**	0.080	−0.264	3.864	0.087	1.542
B. Sc. 4th Year	0.005	−0.793	0.856	0.940	0.156	0.950	6.255	**0.008**	0.139	0.518	4.842	**0.015**	2.287
M. Sc./MS	0.014	−1.030	1.271	0.837	0.196	1.824	9.230	**0.004**	0.207	1.875	7.912	**0.002**	2.962
Current residence													
With family	[Reference]				[Reference]								
Hostel (with friends/roommates)	0.138	0.082	1.683	0.031	−0.077	−4.194	0.961	0.218	−0.074	−3.408	0.794	0.222	2.587
Mess/Rented house with others	0.061	−0.488	1.409	0.340	−0.015	−3.427	2.678	0.810	−0.060	−3.745	1.231	0.321	2.601
Permanent residence													
Urban	[Reference]				[Reference]								
Semi‐Urban	−0.006	−0.714	0.620	0.890	−0.082	−4.095	0.200	0.075	−0.110	−3.945	−0.443	**0.014**	1.405
Rural	0.010	−0.480	0.586	0.845	−0.238	−6.061	−2.629	**<0.001**	−0.205	−4.551	−1.754	**< 0.001**	1.516
Father's educational qualification													
No formal education	[Reference]				[Reference]								
Primary	0.033	−0.935	1.499	0.649	−0.067	−5.787	2.049	0.349	0.008	−3.001	3.386	0.906	3.443
Secondary	−0.067	−1.686	0.755	0.454	0.009	−3.720	4.139	0.916	0.010	−3.014	3.393	0.907	5.123
Higher Secondary	−0.012	−1.341	1.179	0.900	−0.067	−5.519	2.590	0.478	0.016	−3.004	3.605	0.858	5.873
Bachelor's or above	−0.099	−1.847	0.731	0.395	−0.024	−4.602	3.697	0.830	−0.021	−3.708	3.058	0.850	8.635
Mother's educational qualification													
No formal education	[Reference]				[Reference]								
Primary	0.045	−0.985	1.650	0.620	0.082	−2.233	6.248	0.353	−0.020	−3.859	3.055	0.819	5.218
Secondary	0.142	−0.474	2.165	0.208	0.069	−2.889	5.603	0.530	−0.085	−4.849	2.073	0.431	8.146
Higher Secondary	0.285	0.358	3.193	**0.014**	0.026	−4.021	5.106	0.815	−0.098	−5.411	2.029	0.372	8.551
Bachelor's or above	0.209	−0.043	2.936	**0.057**	−0.006	−4.923	4.666	0.958	−0.036	−4.587	3.229	0.733	7.702
Father's employment status													
Employed	[Reference]				[Reference]								
Unemployed	0.033	−0.794	1.792	0.449	0.039	−2.217	6.104	0.359	0.022	−2.485	4.298	0.599	1.182
Retired	0.044	−0.324	0.978	0.324	−0.020	−2.589	1.601	0.643	0.059	−0.511	2.905	0.169	1.290
Business	−0.025	−0.695	0.407	0.608	0.033	−1.158	2.390	0.495	−0.007	−1.561	1.331	0.876	1.559
Mother's employment status													
Employed	[Reference]				[Reference]								
Unemployed	−0.005	−0.783	0.705	0.919	−0.055	−3.893	0.897	0.219	0.005	−1.835	2.069	0.906	1.334
Personal monthly cost													
<5000 BDT	[Reference]				[Reference]								
5000–10,000 BDT	0.033	−0.318	0.731	0.439	0.016	−1.365	2.008	0.708	0.053	−0.472	2.278	0.197	1.183
>10,000 BDT	−0.004	−1.939	1.771	0.929	0.016	−4.798	7.143	0.700	0.047	−1.979	7.755	0.244	1.144
Participant in environmental seminars/workshops													
Yes	[Reference]				[Reference]								
No	0.023	−0.467	0.723	0.672	−0.028	−2.414	1.414	0.608	−0.101	−3.093	0.028	0.054	1.922
Learned about biodegradable packaging in university courses													
Yes	[Reference]				[Reference]								
No	−0.237	−1.927	−0.684	**< 0.001**	−0.127	−4.295	−0.294	0.025	−0.146	−3.842	−0.580	**0.008**	2.103
Participate in a cleanliness drive or environmental volunteer work													
Yes	[Reference]				[Reference]								
No	−0.088	−1.139	0.148	0.131	−0.145	−4.747	−0.604	**0.012**	−0.132	−3.736	−0.358	**0.018**	2.176
Received any formal education or training on environmental conservation													
Yes	[Reference]				[Reference]								
No	−0.121	−1.274	−0.065	**0.030**	−0.074	−3.285	0.604	0.176	−0.116	−3.343	−0.172	**0.030**	1.987
Following any social media pages or influencers promoting eco‐friendly products													
Yes	[Reference]				[Reference]								
No	−0.233	−1.867	−0.717	**< 0.001**	−0.137	−4.333	−0.631	**0.009**	−0.112	−3.211	−0.193	**0.027**	1.788
***R*** ^**2**^	**0.447**				**0.469**				**0.498**				
Adjusted *R* ^2^	**0.398**				**0.421**				**0.453**				

*Note:*Reference” denotes the baseline category against which other categories are compared in the regression models. The reported β coefficients represent the estimated change relative to the reference group. Bold values indicate statistical significance.

Abbreviations:β, unstandardized regression coefficient; CI, confidence interval; LL, lower limit; UL, upper limit; VIF, variation inflation factor.

^a^ Included Christianity and Buddhism.

Table 7 shows the barriers impeding the adoption of biodegradable packaging. The most commonly described concern was the difficulty in finding biodegradable packaging in local markets (14.1%). Moreover, higher cost compared to conventional plastic packaging was identified by 12.1% of respondents, which highlights that affordability is a significant restriction for many consumers. Lack of awareness (10.5%) and inadequate familiarity with biodegradable packaging (9.4%) were also significant obstacles to choosing biodegradable packaging. Furthermore, inadequate waste management infrastructure (9.2%) and social or cultural norms favoring traditional packaging (8.0%) might be factors discourage consumer behavior. shortage of proper disposal methods (7.7%), lack of clear labeling or certification (7.8%), and uncertainties about product quality (7.1%) further illustrated how they hindered trust in biodegradable alternatives. Approximately 6.0% believed the design or appearance of biodegradable packaging is often unattractive, and 6.7% felt such packaging is not durable enough for daily use.

**Table 7:** Barriers of choosing biodegradable packaging.

**Barriers**	**Frequency**	**Percentage** a
Higher cost compared to plastic packaging	250	12.1%
Difficult to find in local markets	292	14.1%
Lack of clear labeling or certification	162	7.8%
Uncertainty about proper disposal methods	160	7.7%
Not sure about the quality	148	7.1%
Inadequate waste management	190	9.2%
Limited knowledge about biodegradable packaging	195	9.4%
Social or cultural norms favoring traditional packaging	165	8.0%
Design or appearance is often unattractive	124	6.0%
Lack of awareness	218	10.5%
Not durable enough for daily use	139	6.7%
Other's	27	1.3%

*Note:* Percentages are calculated based on the total number of responses rather than the total number of participants.

^a^ Multiple responses were allowed.

Table 8 demonstrate the environmental concerns raised by respondents, in which the most alarming environmental concern was pollution of rivers, oceans, and natural areas by plastic packaging (11.9%), followed by contribution of packaging waste to overall environmental degradation (10.0%), potential risk of wildlife due to plastic and non‐biodegradable packaging (9.8%), long‐term health and environmental risks of plastic micro‐particles (9,1%), and finally damage to soil and compost quality due to improper disposal of packaging (9.1%).

**Table 8:** Environmental concerns.

**Concerns**	**Frequency**	**Percentage** a
Plastic packaging pollutes rivers, oceans, and natural areas	323	11.9%
Food packaging waste contributes significantly to overall environmental degradation	273	10.0%
Packaging production increases greenhouse gas emissions	240	8.8%
Most packaging is made from non‐renewable resources	220	8.1%
Improper disposal of packaging harms soil and compost quality	247	9.1%
Wildlife is at risk due to plastic and non‐biodegradable packaging	266	9.8%
Climate change is worsened by the life cycle of traditional packaging	229	8.4%
Limited recycling or composting infrastructure leads to packaging waste buildup	216	7.9%
Lack of innovation in sustainable packaging technologies	223	8.2%
Energy‐intensive processes are used in traditional packaging waste management	184	6.8%
Long‐term health and environmental risks of plastic micro‐particles	247	9.1%
Other's	49	1.8%

*Note:* Percentages are calculated based on the total number of responses rather than the total number of participants.

^a^ Multiple responses were allowed.

## 4 Discussions

The findings from a survey conducted among university students in southern Bangladesh revealed moderate levels of KAP regarding biodegradable packaging. Specifically, students demonstrated an average knowledge score of 54.7%, a positive attitude of 58.9%, and practical engagement of 57%. Significant differences were observed based on demographic factors, with higher levels of KAP identified among students aged 21–23, hostel residents, and educated mothers. Notably, this study highlights the importance of gender and academic progression, with female students and those in advanced years of study showing better attitudes and practices. The barriers identified included low exposure to biodegradable packaging and significant challenges in market accessibility, revealing critical gaps that impede sustainable packaging adoption. These moderate KAP scores are somewhat lower than the high knowledge but only moderate attitude and practice commonly reported among Malaysian university students toward sustainable consumption and environmental sustainability [50, 51, 52]. Similarly, studies among Indonesian and Chinese university students have found relatively high levels of sustainability knowledge alongside low‐to‐moderate levels of pro‐environmental behavior [27, 53]. Although direct comparisons should be interpreted with caution due to differences in study design and measurement tools, our findings suggest that Bangladeshi students may exhibit slightly lower knowledge levels while reflecting a broader regional pattern of an awareness–action gap. To enhance these KAP levels, a focus on targeted educational initiatives within university curricula is essential, along with promoting community engagement programs specifically aimed at improving market accessibility for biodegradable packaging.

In terms of knowledge, a significant predictor associated with higher levels of knowledge was the educational attainment of participants' mothers. Students whose mothers had completed higher secondary education exhibited a better understanding of biodegradable materials. This finding underscores the role of familial educational background in shaping environmental consciousness as children often inherit values and knowledge from their parents. An educated parent is more likely to actively engage in and promote responsible environmental behavior, thereby setting a model for their children to emulate. This pattern is supported by research indicating that parental education is a strong predictor of children's attitudes towards sustainability and environmental responsibility [54, 55]. Additionally, hostel residency emerged as a significant factor: students living in hostels may engage more deeply with peers discussing sustainable practices and resources, thus further enhancing their knowledge base. For instance, one study highlighted that communal living in hostels facilitates the exchange of ideas regarding the usage and conservation of common resources, which directly impacts students' ecological consciousness [56]. Educational institutions can leverage these dynamics by fostering environmental discussions and collaboration among students to broaden their understanding of biodegradable packaging.

The findings revealed gender differences as a significant predictor, with female students displaying more favorable attitudes toward biodegradable packaging than their male counterparts. The gender pattern mirrors the findings from pro‑environmental behavior studies in Europe and Asia, where female students often report stronger environmental concern and more consistent sustainable behaviors [57, 58, 59]. Gender differences play a significant role in eco‐centric behaviors, with women frequently demonstrating more favorable attitudes toward sustainability than men. As noted in the existing literature, gender socialization may account for these disparities, as women tend to internalize the norms surrounding nurturing and accountability for environmental stewardship [60]. Furthermore, higher KAP among senior students is comparable to Malaysian and Indonesian data, where sustainability KAP increase with exposure to university sustainability curricula and green campus initiatives [50, 61, 62]. This indicates a need for universities to act as platforms for fostering positive attitudes through the integration of sustainability into the education system, ensuring that students are not just knowledgeable, but also attuned to the implications of their choices concerning packaging.

In examining the practical engagement of students, the study highlighted that female students and those in the later stages of their studies were more active participants in employing biodegradable packaging options. This aligns with previous studies that illustrate that women generally exhibit greater environmental concerns and willingness to engage in sustainable practices [63]. This trend may be connected to the broader pattern of women generally showing greater concern for environmental issues and being more likely to adopt eco‐friendly practices [64]. Moreover, the study found that students from urban areas exhibited better practical engagement in using biodegradable packaging than their rural counterparts. The higher practical engagement in urban students parallels evidence from India and multi‑country packaging studies (including India and China), where better infrastructure, more product options and clearer information in cities facilitate sustainable packaging behaviors [35, 65]. This finding aligns with various studies indicating that urban residences often provide easier access to sustainable products and initiatives, creating discrepancies in engagement levels across demographics [66]. Addressing these disparities requires tailored strategies to ensure equitable access to educational resources and biodegradable options, particularly in rural areas.

A crucial aspect of students' KAP concerning biodegradable packaging is their exposure to the relevant educational content through university courses. Those who learned about biodegradable materials during their studies exhibited higher knowledge levels, underscoring the importance of integrating sustainability into academic curricula. Research indicates that educational interventions can significantly increase awareness of packaging's environmental impacts, which, in turn, positively influences consumer behavior [67]. Furthermore, active participation in cleanliness drives or environmental volunteer work has been identified as a significant predictor of practical engagement in sustainable practices. Such activities not only provide experiential learning opportunities but also foster a sense of responsibility and community among participants. Studies have shown that involvement in volunteerism enhances environmental sensitivity, leading individuals to adopt more sustainable behaviors [68]. This supports the idea that educational experiences associated with volunteerism can amplify environmental consciousness and sustainable development.

Social media pages and influencers promoting eco‐friendly products have also emerged as significant predictors of students' attitudes toward biodegradable packaging. The strong role of social media and online sources in our sample aligns with Malaysian and Saudi findings, where the internet is a dominant channel for sustainability information among students [25, 62]. Social media play a crucial role in shaping public perceptions and providing ongoing education about sustainability. The curated content from eco‐friendly influencers can raise awareness and challenge misconceptions about biodegradable packaging, thus affecting students' preferences and buying behavior [69]. As younger generations increasingly rely on social media for information, leveraging these platforms for campaigns focused on sustainable packaging can effectively promote positive attitudes and practices toward eco‐friendly products. Additionally, formal education and training in environmental conservation have been found to enhance both knowledge and positive attitudes. Similarly, Malaysian, Indonesian, Chinese and Gulf‐region universities, formal sustainability education and campus programs in Asia consistently show positive effects on students' environmental behavior, although knowledge alone is often insufficient without supportive social and institutional contexts [27, 59, 61, 62]. Such programs can provide participants with critical insights into their ecological footprints, while fostering a sense of agency in driving sustainable consumption [70, 71]. Therefore, embedding formal environmental education into student experience is vital for promoting a more knowledgeable and proactive generation of consumers.

Finally, significant barriers to adopting biodegradable packaging were highlighted, with the accessibility of biodegradable options in the local market being the most frequently cited challenge. Despite demonstrating an awareness of the environmental benefits associated with such materials, the participants faced hurdles in finding them readily available. The market barriers reported by Bangladeshi students resemble constraints documented for eco‑ and sustainable packaging in Malaysia and India, where limited availability, higher prices and confusing information undermine the translation of favorable attitudes into actual purchasing and disposal behavior [72, 73]. Furthermore, student concerns regarding plastic pollution showcased a strong environmental consciousness, but also revealed a gap between awareness and action, as the participants expressed frustration due to the lack of options. To address these overarching issues, initiatives aimed at increasing the availability of biodegradable packaging solutions in local markets should be prioritized. Educational campaigns highlighting the recycling and disposal methods specific to biodegradable materials can further empower consumers to make informed choices, ensuring that the sustainable attributes of these products are well‐communicated [74]. Engaging stakeholders, policymakers, and local businesses in collaborative efforts to enhance the infrastructure for sustainable packaging can bridge the gap between awareness and practical adoption among university students.

## 5 Limitations

Despite providing important insights, this study had several limitations that should be acknowledged. First, the use of a cross‐sectional design limits the ability to establish causal relationships between sociodemographic factors and students' KAP toward biodegradable packaging. Second, reliance on convenience sampling from only two universities in southern Bangladesh may restrict the generalizability of the findings to other higher education institutions across the country. Third, data were collected through self‐reported questionnaires, which may have been subject to recall bias and social desirability bias, potentially leading participants to overstate environmentally friendly practices. Fourth, while the study measured KAP using structured scales, it did not capture deeper qualitative insights into students' motivations, perceptions, and cultural influences, which could enrich the understanding of sustainable behavior. Fifth, our analysis of the barriers and environmental concerns is purely descriptive. Because we did not run formal inferential statistical tests on these specific sections, they should be interpreted as general trends rather than definitive statistical relationships. Additionally, the study did not assess external structural or policy‐level factors such as pricing policies, industry incentives, or government regulations, which might also significantly influence the adoption of biodegradable packaging. Future studies should explore the associations between perceived barriers, environmental concerns, and KAP scores, as well as their relationships with socio‐demographic factors, to better understand the drivers of biodegradable packaging adoption. Finally, as the survey was conducted within a limited 2‐month period, seasonal or temporal variations in consumer behavior could not be examined. Future studies incorporating longitudinal designs, larger and more diverse samples, and mixed‐method approaches would provide more robust evidence and broader applicability of the findings.

## 6 Practical Implications and Future Perspectives

The findings of this study provide several practical implications for improving the biodegradable packaging of KAP among university students in southern Bangladesh. Integrating sustainability concepts into university curricula and offering experiential opportunities, such as volunteerism and cleanliness drives, can strengthen students' environmental knowledge and foster positive attitudes. As female students, hostel residents, and those in advanced study years exhibited higher KAP, targeted interventions should focus on engaging male students, early year cohorts, and those from rural areas who face limited access to biodegradable options. Establishing eco‐shops on campuses, encouraging peer‐led hostel initiatives, and leveraging social media influencers can enhance awareness and practical engagement. Furthermore, the strong influence of maternal education highlights the need to extend sustainability campaigns beyond campuses to families and communities, thereby reinforcing environmentally responsible values at home. To overcome the barrier of limited market accessibility, collaboration between universities, policymakers, and local businesses is crucial for expanding the availability and affordability of biodegradable packaging. Together, these strategies can bridge the gap between awareness and adoption, and position universities as central actors in driving sustainable consumption practices in Bangladesh.

Future research should build on the present findings by examining the interrelationships between KAP, perceived barriers, and environmental concerns to better understand the mechanisms driving biodegradable packaging adoption. Given the observed influence of gender, academic level, residence, and parental education, future studies should further explore how these socio‐demographic factors shape sustainable behavior using longitudinal and mixed‐method approaches. Additionally, the identified gap between awareness and actual practice suggests a need to investigate behavioral and structural constraints more deeply, particularly the roles of cost, market availability, and waste management infrastructure. Targeted strategies should also be developed for groups with comparatively lower engagement, such as early‐year students and those from rural backgrounds. Furthermore, enhancing access to biodegradable packaging through policy support, improved supply chains, and clearer labeling systems should be prioritized. Overall, integrating educational, behavioral, and structural approaches will be essential to translate positive attitudes into consistent sustainable practices among university students in Bangladesh.

## 7 Conclusions

This study is the first to explore KAP, including barriers to adopting biodegradable packages, along with environmental concerns among university students in Bangladesh. findings of the study revealed a significant response rate for the test of knowledge, attitude, and practice of 54.7%, 58.9%, and 57%, respectively, which is yet not satisfactory. Various factors such as gender, educational level, and parents' educational background, were strongly associated with these factors. However, bridging the gap between positive attitudes and actual practices of biodegradable packaging could be a solution. Moreover, academic engagement in sustainable environmental solutions and volunteer participation in various programs addressing eco‐friendly packaging solutions were key indicators influencing participants' preference for biodegradable packaging over plastic packaging. However, limited accessibility and higher costs compared to plastics are significant barriers. Environmental concerns, such as pollution of nature and rivers due to plastic and their non‐disposable nature, are alarming concerns to the participants. The results of the study emphasize that young people who are being considered as future decision makers can break this barrier with the help of supportive campaigns and policy intervention. Additionally, industrialists and local markets should improve the accessibility and affordability of biodegradable packaging. Future research should explore the fundamental barriers of cost, availability through marketplaces, strengthening academic learning, social media campaigns, policy support, etc., to discover a convenient, eco‐friendly packaging solution nationwide.

## Author Contributions

**Razia Sultana:** conceptualization, methodology, data curation, investigation, validation, visualization, resources, writing – original draft, writing – review and editing. **Jannatun Naeem:** methodology, data curation, investigation, validation, writing – original draft, writing – review and editing. **Nadi Rani Dey:** methodology, data curation, investigation, writing – review and editing. **Maliha Tayaba:** methodology, data curation, investigation, writing – review and editing. **Sultan Mahmud Imran:** methodology, software, data curation, formal analysis, writing – review and editing. **Nitai Roy:** conceptualization, methodology, software, investigation, validation, supervision, visualization, project administration, resources, writing – review and editing, writing – original draft.

## Funding

The authors have nothing to report.

## Ethics Statement

This study was conducted in accordance with the ethical principles outlined in the Declaration of Helsinki (2013). Prior to data collection, ethical clearance was obtained from the appropriate institutional authorities. Specifically, ethical approval was granted by the Institutional Ethical Committee of Patuakhali Science & Technology University (Approval No. PSTU/IEC/2025/20 (iii)). Participation in the study was entirely voluntary. All participants were informed about the purpose and procedures of the research, and informed consent was obtained before administering the survey. Respondents were assured of the confidentiality and anonymity of their information, and no personally identifiable data were collected. Participants were also informed of their right to withdraw from the study at any time without any consequences.

## Conflicts of Interest

The authors declare no conflicts of interest.

## 1 Transparency Statement

Mst. Razia Sultana affirms that this manuscript is an honest, accurate, and transparent account of the study being reported; that no important aspects of the study have been omitted; and that any discrepancies from the study as planned have been explained.

## Supporting information


Supporting File 1

Supporting File 2

## Data Availability

The data that support the findings of this study are available on request from the corresponding author. The data are not publicly available due to privacy or ethical restrictions.
