# Supplementary material for: Exploring Knowledge, Attitudes, Practices, Environmental Concerns, and Barriers to Biodegradable Packaging Among University Students at Two Public Universities in Bangladesh: A Cross‐Sectional Study
Source: Health Sci Rep. 2026 Jul 30;9(8):e72880. doi: 10.1002/hsr2.72880 (PMC13421799; doi:10.1002/hsr2.72880)
Supplement: Supplementary file 1 — Supporting File 1 [file HSR2-9-e72880-s001.pdf]

"বাংলাদেশের বিশ্ববিদ্যালয়ের শিক্ষার্থীদের মধ্যে জৈব-পচনশীল প্যাকেজিংয়ের জ্ঞান, মনোভাব, অনুশীলন, পরিবেশগত উদ্বেগ এবং বাধা অনুসন্ধান" শীর্ষক একটি গবেষণা

মডিউল ১: জনসংখ্যাতাত্ত্বিক এবং প্রাসঙ্গিক তথ্য

১. বয়স (বছরে):	.... বছর
২. লিঙ্গ:	☐ পুরুষ ☐ নারী
৩. ধর্ম:	☐ ইসলাম ☐ হিন্দুধর্ম ☐ অন্যান্য: ___________
৪. বর্তমান শিক্ষাগত স্তর:	☐ বি.এসসি ১ম বর্ষ ☐ ২য় বর্ষ ☐৩য় বর্ষ ☐ ৪র্থ বর্ষ ☐ এম.এসসি/এম.এস
৫. বর্তমান বসবাসের ঠিকানা:	☐ পরিবারের সাথে ☐ হোস্টেলে ☐ মেস/ভাড়াবাড়ি
৬. স্থায়ী ঠিকানা:	☐ শহর ☐ আধা-শহর ☐ গ্রাম
৭. পিতার শিক্ষাগত যোগ্যতা:	☐ নেই ☐ প্রাথমিক ☐ মাধ্যমিক ☐ উচ্চ মাধ্যমিক ☐ স্নাতক বা তদূর্ধ্ব
৮. মাতার শিক্ষাগত যোগ্যতা:	☐ নেই ☐ প্রাথমিক ☐ মাধ্যমিক ☐ উচ্চ মাধ্যমিক ☐ স্নাতক বা তদূর্ধ্ব
৯. পিতার পেশা:	☐ চাকরিজীবী ☐ বেকার ☐ অবসরপ্রাপ্ত ☐ ব্যবসায়ী
১০. মাতার পেশা:	☐ চাকরিজীবী ☐ বেকার ☐ গৃহিণী ☐ অবসরপ্রাপ্ত
১১. পারিবারিক মাসিক আয় (BDT):	.... টাকা
১১. ব্যক্তিগত মাসিক খরচ (BDT):	.... টাকা
১২. আপনি কি পরিবেশ বিষয়ক কোন সেমিনার/ওয়ার্কশপে অংশগ্রহণ করেছেন?	☐ হ্যাঁ ☐ না
১৩. আপনি কি বিশ্ববিদ্যালয়ের কোন কোর্সে জৈব-পচনশীল প্যাকেজিং (যেমন পাটজাত ব্যাগ, পেপার বা কাগজের ব্যাগ ইত্যাদি) সম্পর্কে শিখেছেন?	☐ হ্যাঁ ☐ না
১৪. আপনি কি কখনো পরিচ্ছন্নতা বা পরিবেশ সংক্রান্ত স্বেচ্ছাসেবী কর্মকাণ্ডে অংশ নিয়েছেন?	☐ হ্যাঁ ☐ না
১৫.আপনি কি পরিবেশ রক্ষা বিষয়ক কোনো প্রশিক্ষণ বা শিক্ষা পেয়েছেন?	☐ হ্যাঁ ☐ না
১৬. আপনি কি পরিবেশবান্ধব পণ্য প্রচারকারী কোন সোশ্যাল মিডিয়া বা প্রভাবশালীদের অনুসরণ করেন?	☐ হ্যাঁ ☐ না
১৭. আপনি প্রথম কোথায় জৈব-পচনশীল প্যাকেজিং সম্পর্কে জেনেছেন?	☐টিভি ☐পত্রিকা ☐সোশ্যাল মিডিয়া ☐ বন্ধু/পরিবার ☐একাডেমিক/কাজের পরিবেশ ☐ অন্যান্য: ___________

মডিউল ২: জৈব-পচনশীল প্যাকেজিং সম্পর্কে জ্ঞান

(আপনার বর্তমান জ্ঞানের উপর ভিত্তি করে নিম্নলিখিত প্রশ্নের উত্তর দিন।)

বিবৃতি	মন্তব্য
১. আপনি কি কখনো "জৈব-পচনশীল খাদ্য প্যাকেজিং" (যেমন পাটজাত ব্যাগ, পেপার বা কাগজের ব্যাগ ইত্যাদি) শব্দটি শুনেছেন?	☐ হ্যাঁ ☐ না ☐ নিশ্চিত না
২. আপনি কি জানেন এটি সাধারণত উদ্ভিজ্জ উপাদান যেমন কর্নস্টার্চ বা সেলুলোজ দিয়ে তৈরি হয়?	☐ হ্যাঁ ☐ না ☐ নিশ্চিত না
৩. দোকানে কেনাকাটা করার সময় আপনি কি জৈব-পচনশীল প্যাকেজিং সনাক্ত করতে পারেন?	☐ হ্যাঁ ☐ না ☐ নিশ্চিত না
৪. আপনি কি মনে করেন জৈব-পচনশীল প্যাকেজিং পরিবেশের জন্য ক্ষতিকর	☐ হ্যাঁ ☐ না ☐ নিশ্চিত না
৫. আপনি কি জানেন জৈব-পচনশীল পরিবেশে স্বাভাবিকভাবে পচে যায়?	☐ হ্যাঁ ☐ না ☐ নিশ্চিত না
৬. জৈব-পচনশীল প্যাকেজিং পচন হতে কি প্লাস্টিকের তুলনায় বেশি সময় নেয়?	☐ হ্যাঁ ☐ না ☐ নিশ্চিত না
৭. আপনি কি জানেন যে বায়োডিগ্রেডেবল প্যাকেজিং উপযুক্ত পরিস্থিতিতে কম্পোস্ট করা যেতে পারে?	☐ হ্যাঁ ☐ না ☐ নিশ্চিত না
৮. আপনি কি এমন কোন প্রতীক বা সার্টিফিকেশনের সাথে পরিচিত যা জৈব-পচনশীল প্যাকেজিং নির্দেশ করে?	☐ হ্যাঁ ☐ না ☐ নিশ্চিত না
৯. আপনি কি জানেন যে জৈব-পচনশীল উপকরণ ব্যবহার পরিবেশগত স্থায়িত্বের লক্ষ্যগুলিকে সমর্থন করে?	☐ হ্যাঁ ☐ না ☐ নিশ্চিত না
১০. আপনি কি জানেন যে জৈব-পচনশীল প্যাকেজিং কখনও কখনও প্লাস্টিকের প্যাকেজিংয়ের চেয়ে বেশি দামের হতে পারে?	☐ হ্যাঁ ☐ না ☐ নিশ্চিত না
১১. "জৈব-পচনশীল বা বায়োডিগ্রেডেবল " লেবেলযুক্ত সমস্ত পণ্য যেকোনো পরিবেশে দ্রুত পচে যায়।	☐ হ্যাঁ ☐ না ☐ নিশ্চিত না
১২. কলাপাতা বা মাটির পাত্রের মতো ঐতিহ্যবাহী উপাদানে জৈব- পচনশীল প্যাকেজিং তৈরি করা যায় না — আপনি কি এতে বিশ্বাস করেন?	☐ হ্যাঁ ☐ না ☐ নিশ্চিত না

মডিউল ৩: জৈব-পচনশীল প্যাকেজিংয়ের প্রতি মনোভাব

(আপনার মনোভাব সম্পর্কে নিম্নলিখিত বিবৃতিগুলির সাথে আপনি কতটা একমত বা অসম্মত তা অনুগ্রহ করে নির্দেশ করুন)।

বিবৃতি	মন্তব্য
১. কেনাকাটার সময় আমি প্যাকেজিংয়ের পরিবেশগত প্রভাব নিয়ে ভাবি	☐ দৃঢ়ভাবে সম্মত ☐ একমত ☐ নিরপেক্ষ ☐ অসম্মত ☐ দৃঢ়ভাবে অসম্মত
২. আমি বাংলাদেশের প্লাস্টিক দূষণ নিয়ে চিন্তিত	☐ দৃঢ়ভাবে সম্মত ☐ একমত ☐ নিরপেক্ষ ☐ অসম্মত ☐ দৃঢ়ভাবে অসম্মত
৩. মূল্য বেশি হলেও আমি জৈব-পচনশীল প্যাকেজিংকেই প্রাধান্য দেই	☐ দৃঢ়ভাবে সম্মত ☐ একমত ☐ নিরপেক্ষ ☐ অসম্মত ☐ দৃঢ়ভাবে অসম্মত
৪. আমি মনে করি জৈব-পচনশীল প্যাকেজিং স্বাস্থ্যঝুঁকি কমাতে পারে না	☐ দৃঢ়ভাবে সম্মত ☐ একমত ☐ নিরপেক্ষ ☐ অসম্মত ☐ দৃঢ়ভাবে অসম্মত
৫. পরিবেশবান্ধব প্যাকেজিং দেখতে আকর্ষণীয় নয়	☐ দৃঢ়ভাবে সম্মত ☐ একমত ☐ নিরপেক্ষ ☐ অসম্মত ☐ দৃঢ়ভাবে অসম্মত
৬. জৈব-পচনশীল প্যাকেজিং সহ পণ্য নির্বাচন করার জন্য আমি দায়ী বোধ করি	☐ দৃঢ়ভাবে সম্মত ☐ একমত ☐ নিরপেক্ষ ☐ অসম্মত ☐ দৃঢ়ভাবে অসম্মত
৭. আমি এ ধরনের পণ্যের জন্য বেশি দাম দিতে রাজি নই	☐ দৃঢ়ভাবে সম্মত ☐ একমত ☐ নিরপেক্ষ ☐ অসম্মত ☐ দৃঢ়ভাবে অসম্মত
৮. বাংলাদেশে জৈব-পচনশীল প্যাকেজিং বাধ্যতামূলক করার দরকার নেই	☐ দৃঢ়ভাবে সম্মত ☐ একমত ☐ নিরপেক্ষ ☐ অসম্মত ☐ দৃঢ়ভাবে অসম্মত
৯. আমি বিশ্বাস করি শিল্পপ্রতিষ্ঠানগুলোকে জৈব-পচনশীল প্যাকেজিং গ্রহণে নেতৃত্ব দেওয়া উচিত	☐ দৃঢ়ভাবে সম্মত ☐ একমত ☐ নিরপেক্ষ ☐ অসম্মত ☐ দৃঢ়ভাবে অসম্মত
১০. আমি মনে করি জৈব-পচনশীল প্যাকেজিং একটি সাময়িক ট্রেন্ড যেটার ভবিষ্যতে তেমন গ্রহণযোগ্যতা বাড়বে না	☐ দৃঢ়ভাবে সম্মত ☐ একমত ☐ নিরপেক্ষ ☐ অসম্মত ☐ দৃঢ়ভাবে অসম্মত
১১. আমি অন্যদের জৈব-পচনশীল প্যাকেজিং প্যাকেজিং ব্যবহারে উৎসাহ দিই	☐ দৃঢ়ভাবে সম্মত ☐ একমত ☐ নিরপেক্ষ ☐ অসম্মত ☐ দৃঢ়ভাবে অসম্মত
১২. শিক্ষার্থীদের জৈব-পচনশীল প্যাকেজিং সম্পর্কে শিক্ষিত করা উচিত	☐ দৃঢ়ভাবে সম্মত ☐ একমত ☐ নিরপেক্ষ ☐ অসম্মত ☐ দৃঢ়ভাবে অসম্মত
১৩. জৈব-পচনশীল প্যাকেজিং ব্যবহার টেকসই ভবিষ্যতের জন্য অবদান রাখে না	☐ দৃঢ়ভাবে সম্মত ☐ একমত ☐ নিরপেক্ষ ☐ অসম্মত ☐ দৃঢ়ভাবে অসম্মত
১৪. জৈব-পচনশীল প্যাকেজিং ব্যবহার করা সকলের অভ্যাস হওয়া উচিত	☐ দৃঢ়ভাবে সম্মত ☐ একমত ☐ নিরপেক্ষ ☐ অসম্মত ☐ দৃঢ়ভাবে অসম্মত

মডিউল ৪: জৈব-পচনশীল প্যাকেজিংয়ের অনুশীলন

(আপনি কত ঘন ঘন নিম্নলিখিত অনুশীলনগুলিতে নিযুক্ত হন তা অনুগ্রহ করে নির্দেশ করুন)

বিবৃতি	মন্তব্য
১. কেনাকাটা করার সময় প্লাস্টিকের ব্যাগ এড়াতে আমি একটি পুনর্ব্যবহারযোগ্য ব্যাগ বহন করি।	☐ কখনো না ☐ খুব কম ☐ মাঝে মাঝে ☐প্রায়ই ☐ সবসময়
২. খাবার বা পণ্য কেনার সময় আমি জৈব-পচনশীল প্যাকেজিং খুঁজি।	☐ কখনো না ☐ খুব কম ☐ মাঝে মাঝে ☐প্রায়ই ☐ সবসময়
৩. আমি প্যাকেজিং উপকরণ পুনঃব্যবহার করি।	☐ কখনো না ☐ খুব কম ☐ মাঝে মাঝে ☐প্রায়ই ☐ সবসময়
৪. আমি পণ্য কেনার সময় প্যাকেজিং এর গায়ে লেবেল দেখে জৈব-অবক্ষয়যোগ্য কিনা তা যাচাই করি।	☐ কখনো না ☐ খুব কম ☐ মাঝে মাঝে ☐প্রায়ই ☐ সবসময়
৫. আমি প্যাকেজিং বর্জ্য সঠিকভাবে ফেলার চেষ্টা করি।	☐ কখনো না ☐ খুব কম ☐মাঝে মাঝে ☐ প্রায়ই ☐ সবসময়
৬. আমি পরিবার বা বন্ধুর সঙ্গে জৈব-পচনশীল প্যাকেজিং এর গুরুত্ব নিয়ে আলোচনা করি।	☐ কখনো না ☐খুব কম ☐ মাঝে মাঝে ☐ প্রায়ই ☐ সবসময়
৭. আমি টেকসই প্যাকেজিং নিয়ে পরিবেশ বিষয়ক কর্মকাণ্ডে অংশ নিই।	☐ কখনো না ☐খুব কম ☐ মাঝে মাঝে ☐ প্রায়ই ☐ সবসময়
৮. আমি প্লাস্টিক প্যাকেজিং পছন্দ করি কারণ এটি বেশি সুবিধাজনক।	☐ কখনো না ☐খুব কম ☐ মাঝে মাঝে ☐ প্রায়ই ☐ সবসময়
৯. আমি এমন খাদ্য সরবরাহ পরিষেবা (ডেলিভারি সার্ভিস) বেছে নিই যারা পরিবেশবান্ধব প্যাক ব্যবহার করে।	☐ কখনো না ☐ খুব কম ☐ মাঝে মাঝে ☐প্রায়ই ☐ সবসময়
১০. আমি মনে করি আমার ব্যক্তিগত পছন্দগুলি প্যাকেজিং বর্জ্যের সমস্যাগুলিকে প্রভাবিত করে না।	☐ কখনো না ☐খুব কম ☐মাঝে মাঝে ☐ প্রায়ই ☐সবসময়
১১. আমি জৈব-পচনশীল প্যাকেজিং বিষয়ক জ্ঞানার্জনের চেষ্টা করি।	☐কখনো না ☐ খুব কম ☐ মাঝে মাঝে ☐প্রায়ই ☐সবসময়
১২. টেকসই প্যাকেজিং সম্পর্কে সচেতনতা বাড়াতে আমি সোশ্যাল মিডিয়া বা অনলাইন প্ল্যাটফর্মে পোস্ট করি।	☐ কখনো না ☐ খুব কম ☐ মাঝে মাঝে ☐প্রায়ই ☐ সবসময়
১৩. টেকসই প্যাকেজিং ব্যবহার আমার কাছে কঠিন বলে মনে হয়।	☐ কখনো না ☐ খুব কম ☐ মাঝে মাঝে ☐প্রায়ই ☐ সবসময়
১৪. আমি দামের উপর ভিত্তি করে পণ্য নির্বাচন করি, প্যাকেজিংয়ের স্থায়িত্বের উপর নয়।	☐ কখনো না ☐ খুব কম ☐মাঝে মাঝে ☐প্রায়ই ☐ সবসময়
১৫. আমি বিশ্ববিদ্যালয়ের সেমিনার, কর্মশালা, অথবা টেকসই প্যাকেজিং সম্পর্কিত প্রচারণায় অংশগ্রহণ করি।	☐ কখনো না ☐ খুব কম ☐ মাঝে মাঝে ☐প্রায়ই ☐ সবসময়

মডিউল ৫: জৈব-পচনশীল প্যাকেজিং গ্রহণে প্রতিবন্ধকতা

জৈব-পচনশীল খাদ্য প্যাকেজিং ব্যবহারে আপনার কী কী চ্যালেঞ্জের মুখোমুখি হতে হয়?<br>**(প্রযোজ্য উত্তর সমূহে টিক দিন)**	A. প্লাস্টিকের তুলনায় ব্যয়বহুল<br>B. স্থানীয় বাজারে সহজে পাওয়া যায় না<br>C. সুস্পষ্ট লেবেল বা সার্টিফিকেশন নেই<br>D. সঠিকভাবে ফেলতে হবে কীভাবে, তা নিয়ে অনিশ্চয়তা<br>E. মান নিয়ে সন্দেহ<br>F. পর্যাপ্ত বর্জ্য ব্যবস্থাপনা নেই<br>G. এই সম্পর্কে জ্ঞানের অভাব<br>H. সামাজিক/সাংস্কৃতিকভাবে প্রচলিত প্যাকেজিং ব্যবহারে চাপ<br>I. ডিজাইন বা চেহারায় তেমন আকর্ষণ নেই<br>J. সচেতনতার অভাব<br>K. দৈনন্দিন ব্যবহারের জন্য যথেষ্ট টেকসই নয়<br>L. অন্যান্য (উল্লেখ করুন): ______________

মডিউল ৬: পরিবেশ সংক্রান্ত উদ্বেগ (প্যাকেজিংয়ের পরিবেশগত প্রভাব সম্পর্কে উদ্বেগ)

নিম্নোক্ত পরিবেশগত বিষয়গুলোর মধ্যে কোনগুলো আপনার খাদ্য প্যাকেজিং নির্বাচনকে প্রভাবিত করে? **(প্রযোজ্য উত্তর সমূহে টিক দিন)**	☐ প্লাস্টিক প্যাকেজিং নদী, সাগর ও প্রকৃতি দূষণ করে<br>☐ খাদ্য প্যাকেজিংয়ের বর্জ্য পরিবেশগত দূষণে উল্লেখযোগ্যভাবে অবদান রাখে<br>☐ প্যাকেজিং উৎপাদনে গ্রীনহাউজ গ্যাস নির্গমন বাড়ে<br>☐ অধিকাংশ প্যাকেজিং অ-পুনঃনবীকরণযোগ্য/বার বার ব্যবহার করা যায় না এমন উপাদান থেকে তৈরি<br>☐ প্যাকেজিংয়ের অনুপযুক্ত নিষ্কাশন মাটি এবং কম্পোস্টের গুণমানের ক্ষতি করে<br>☐ প্লাস্টিক এবং অপচনশীল প্যাকেজিংয়ের কারণে বন্যপ্রাণী ঝুঁকির মধ্যে রয়েছে<br>☐ প্রচলিত প্যাকেজিংয়ের জীবনচক্র জলবায়ু পরিবর্তনকে ত্বরান্বিত করে<br>☐অপর্যাপ্ত রিসাইক্লিং বা কম্পোস্টিং অবকাঠামো প্যাকেজিং বর্জ্য তৈরিতে সহয়তা করে<br>☐ টেকসই প্যাকেজিং প্রযুক্তিতে উদ্ভাবনের অভাব<br>☐ প্রচলিত প্যাকেজিং বর্জ্য ব্যবস্থাপনায় অধিক শক্তি ব্যবহার হয়<br>☐ প্লাস্টিক মাইক্রোপার্টিকেল স্বাস্থ্য ও পরিবেশের জন্য দীর্ঘমেয়াদে ক্ষতিকর<br>☐ অন্যান্য (উল্লেখ করুন): ______________
