# Supplementary material for: Exploring Knowledge, Attitudes, Practices, Environmental Concerns, and Barriers to Biodegradable Packaging Among University Students at Two Public Universities in Bangladesh: A Cross‐Sectional Study
Source: Health Sci Rep. 2026 Jul 30;9(8):e72880. doi: 10.1002/hsr2.72880 (PMC13421799; doi:10.1002/hsr2.72880)
Supplement: Supplementary file 2 — Supporting File 2 [file HSR2-9-e72880-s002.docx]

**Exploring Knowledge, Attitudes, Practices, Environmental Concerns, and Barriers to Biodegradable Packaging Among Bangladeshi University Students**

**Module 1: Demographic and Contextual Information**

| 1. Age (in years) | …………………..years |
| --- | --- |
| 2. Gender | ☐ Male ☐ Female |
| 3. Religion | ☐ Islam ☐ Hinduism ☐ Other:___________ |
| 4. Current educational level | ☐ B.Sc 1st Year ☐ B.Sc 2nd Year ☐ B.Sc 3rd Year  ☐ B.Sc 4th Year ☐ M.Sc/MS |
| 5. Current living arrangement | ☐ With Family  ☐ Hostel (with friends/roommates)  ☐ Mess/Rented house with others |
| 6. Permanent residence | ☐ Urban  ☐ Semi-Urban  ☐ Rural |
| 7. Father’s education level | ☐ No Formal Education ☐ Primary ☐ Secondary  ☐ Higher Secondary ☐ Bachelor’s or Above |
| 8. Mother’s education level | ☐ No Formal Education ☐ Primary ☐ Secondary  ☐ Higher Secondary ☐ Bachelor’s or Above |
| 9. Father’s employment status | ☐ Employed ☐ Unemployed ☐ Retired ☐ Business |
| 10. Mother’s employment status | ☐ Employed ☐ Unemployed ☐ Homemaker ☐ Retired |
| 11. Your monthly personal expenditure (BDT) | ……………………Taka |
| 12. History of participation in environmental seminars/workshops | ☐ Yes ☐ No |
| 13. Have you learned about biodegradable packaging in your university courses? | ☐ Yes ☐ No |
| 14. Have you ever participated in a cleanliness drive or environmental volunteer work? | ☐ Yes ☐ No |
| 15. Have you received any formal education or training on environmental conservation? | ☐ Yes ☐ No |
| 16. Have you followed any social media pages or influencers promoting eco-friendly products? | ☐ Yes ☐ No |
| 17. Where did you first learn about it? | ☐ Television/Radio ☐ Newspaper  ☐ Social media ☐ Friends/Family  ☐ Academic or work setting ☐ Other (please specify)………. |

**Module 2: Knowledge of Biodegradable Packaging** (Please answer the following questions based on your current knowledge.)

| **Statements** | **Response Options** |
| --- | --- |
| 1. Have you ever heard the term “biodegradable food packaging” | ☐ Yes  ☐ No  ☐ Not Sure |
| 2. Are you aware that biodegradable packaging is often made from plant-based materials like cornstarch or cellulose? | ☐ Yes  ☐ No  ☐ Not Sure |
| 3. Can you identify biodegradable packaging when shopping at a store? | ☐ Yes  ☐ No  ☐ Not Sure |
| 4. Biodegradable packaging is harmful to the environment? | ☐ Yes  ☐ No  ☐ Not Sure |
| 5. Do you know that biodegradable packaging breaks down naturally in the environment? | ☐ Yes  ☐ No  ☐ Not Sure |
| 6. Biodegradable packaging takes longer to decompose than plastic. | ☐ Yes  ☐ No  ☐ Not Sure |
| 7. Do you know that biodegradable packaging can be composted under suitable conditions? | ☐ Yes  ☐ No  ☐ Not Sure |
| 8. Are you familiar with any symbols or certifications that indicate biodegradable packaging? | ☐ Yes  ☐ No  ☐ Not Sure |
| 9. Do you know that using biodegradable materials supports environmental sustainability goals? | ☐ Yes  ☐ No  ☐ Not Sure |
| 10. Do you know that biodegradable packaging can sometimes cost more than plastic packaging? | ☐ Yes  ☐ No  ☐ Not Sure |
| 11. All products labeled “biodegradable” decompose quickly in any environment. | ☐ Yes  ☐ No  ☐ Not Sure |
| 12. Biodegradable packaging cannot be made from traditional materials like banana leaves or earthen pots. | ☐ Yes  ☐ No  ☐ Not Sure |

# Module 3: Attitude Toward Biodegradable Packaging (Please indicate how strongly you agree or disagree with the following statements regarding your attitude).

| **Statements** | **Response Options** |
| --- | --- |
| 1. I care about the environmental impact of packaging when shopping. | ☐ Strongly Agree ☐ Agree ☐ Neutral ☐ Disagree ☐ Strongly Disagree |
| 2. I am concerned about plastic pollution in Bangladesh. | ☐ Strongly Agree ☐ Agree ☐ Neutral ☐ Disagree ☐ Strongly Disagree |
| 3. I prefer eco-friendly packaging even if the price is high. | ☐ Strongly Agree ☐ Agree ☐ Neutral ☐ Disagree ☐ Strongly Disagree |
| 4. I believe using biodegradable packaging cannot reduce health risks. | ☐ Strongly Agree ☐ Agree ☐ Neutral ☐ Disagree ☐ Strongly Disagree |
| 5. Eco-friendly packaging is not visually appealing and attractive | ☐ Strongly Agree ☐ Agree ☐ Neutral ☐ Disagree ☐ Strongly Disagree |
| 6. I feel responsible for choosing products with biodegradable packaging. | ☐ Strongly Agree ☐ Agree ☐ Neutral ☐ Disagree ☐ Strongly Disagree |
| 7. I am not willing to pay more for products with biodegradable packaging. | ☐ Strongly Agree ☐ Agree ☐ Neutral ☐ Disagree ☐ Strongly Disagree |
| 8. It is not necessary to make Biodegradable packaging mandatory in Bangladesh. | ☐ Strongly Agree ☐ Agree ☐ Neutral ☐ Disagree ☐ Strongly Disagree |
| 9. I believe industries should take the lead in adopting biodegradable packaging | ☐ Strongly Agree ☐ Agree ☐ Neutral ☐ Disagree ☐ Strongly Disagree |
| 10. I believe biodegradable packaging is a trend that will not grow in the future. | ☐ Strongly Agree ☐ Agree ☐ Neutral ☐ Disagree ☐ Strongly Disagree |
| 11. I encourage others to use biodegradable packaging. | ☐ Strongly Agree ☐ Agree ☐ Neutral ☐ Disagree ☐ Strongly Disagree |
| 12. Students should be educated about biodegradable packaging. | ☐ Strongly Agree ☐ Agree ☐ Neutral ☐ Disagree ☐ Strongly Disagree |
| 13. Using biodegradable packaging does not contribute to a sustainable future. | ☐ Strongly Agree ☐ Agree ☐ Neutral ☐ Disagree ☐ Strongly Disagree |
| 14. Using biodegradable packaging should be a habit for everyone. | ☐ Strongly Agree ☐ Agree ☐ Neutral ☐ Disagree ☐ Strongly Disagree |

**Module 4: Practice of biodegradable packaging**

Please indicate how frequently you engage in the following practices.

| **Statements** | **Response Options** |
| --- | --- |
| 1. I carry a reusable bag to avoid plastic bags while shopping. | ☐ Never ☐ Rarely ☐ Sometimes ☐ Often ☐ Always |
| 2. I look for biodegradable packaging when buying food or products. | ☐ Never ☐ Rarely ☐ Sometimes ☐ Often ☐ Always |
| 3. I reuse packaging materials instead of throwing them away. | ☐ Never ☐ Rarely ☐ Sometimes ☐ Often ☐ Always |
| 4. I check product labels to see if the packaging is biodegradable. | ☐ Never ☐ Rarely ☐ Sometimes ☐ Often ☐ Always |
| 5. I dispose of packaging waste responsibly. | ☐ Never ☐ Rarely ☐ Sometimes ☐ Often ☐ Always |
| 6. I talk to friends or family about using biodegradable packaging. | ☐ Never ☐ Rarely ☐ Sometimes ☐ Often ☐ Always |
| 7. I participate in environmental activities related to sustainable packaging. | ☐ Never ☐ Rarely ☐ Sometimes ☐ Often ☐ Always |
| 8. I prefer plastic packaging because it is more convenient. | ☐ Never ☐ Rarely ☐ Sometimes ☐ Often ☐ Always |
| 9. I choose food delivery services that offer biodegradable containers. | ☐ Never ☐ Rarely ☐ Sometimes ☐ Often ☐ Always |
| 10. I believe my personal choices don’t impact packaging waste issues. | ☐ Never ☐ Rarely ☐ Sometimes ☐ Often ☐ Always |
| 11. I make an effort to educate myself on biodegradable packaging practices. | ☐ Never ☐ Rarely ☐ Sometimes ☐ Often ☐ Always |
| 12. I post on social media or online platforms to raise awareness about sustainable packaging. | ☐ Never ☐ Rarely ☐ Sometimes ☐ Often ☐ Always |
| 13. I find sustainable packaging options too difficult to use. | ☐ Never ☐ Rarely ☐ Sometimes ☐ Often ☐ Always |
| 14. I choose products based on price, not packaging sustainability. | ☐ Never ☐ Rarely ☐ Sometimes ☐ Often ☐ Always |
| 15. I attend university seminars, workshops, or campaigns related to packaging sustainability. | ☐ Never ☐ Rarely ☐ Sometimes ☐ Often ☐ Always |

**Module 5: Barriers of choosing biodegradable packaging**

| What challenges do you face in using biodegradable food packaging? (Please select all that apply) | 1. Higher cost compared to plastic packaging 2. Difficult to find in local markets 3. Lack of clear labeling or certification 4. Uncertainty about proper disposal methods 5. Not sure about the quality 6. Inadequate waste management 7. Limited knowledge about biodegradable packaging 8. Social or cultural norms favoring traditional packaging 9. Design or appearance is often unattractive 10. Lack of awareness 11. Not durable enough for daily use 12. Other (please specify): ____________ |
| --- | --- |

**Module 6: Environmental Concerns (Concerns about environmental impacts of packaging)**

| **Which of the following environmental concerns influence your views about food packaging? (Select all that apply)** | **☐ Plastic packaging pollutes rivers, oceans, and natural areas**  **☐ Food packaging waste contributes significantly to overall environmental degradation**  **☐ Packaging production increases greenhouse gas emissions**  **☐ Most packaging is made from non-renewable resources**  **☐ Improper disposal of packaging harms soil and compost quality**  **☐ Wildlife is at risk due to plastic and non-biodegradable packaging**  **☐ Climate change is worsened by the life cycle of traditional packaging**  **☐ Limited recycling or composting infrastructure leads to packaging waste buildup**  **☐ Lack of innovation in sustainable packaging technologies**  **☐ Energy-intensive processes are used in traditional packaging waste management**  **☐ Long-term health and environmental risks of plastic micro-particles**  **☐ Other (please specify): ___________** |
| --- | --- |
